# Importance and benefits of the doctoral thesis for medical graduates

**DOI:** 10.3205/zma001007

**Published:** 2016-02-15

**Authors:** Marianne Giesler, Martin Boeker, Götz Fabry, Silke Biller

**Affiliations:** 1 University of Freiburg, Medical Faculty, Office of Student Affairs, Centre for Evaluation of Teaching in Medicine Baden-Württemberg, Freiburg, Germany; 2 University of Freiburg, Medical Faculty, Department for Medical Biometry and Medical Informatics, Freiburg, Germany; 3 University of Freiburg, Medical Faculty, Department for Medical Psychology and Sociology, Freiburg, Germany; 4 University Basel, Medical Faculty, Office of Student Affairs, Basel, Switzerland

**Keywords:** Doctorate, medical, Methods, academic, Graduate survey, Motivation, Evaluation

## Abstract

**Introduction: **The majority of medical graduates in Germany complete a doctorate, even though a doctoral degree is not necessary for the practice of medicine. So far, little is known about doctoral candidates’ view on the individual benefit a doctoral thesis has for them. Consequently, this is the subject of the present investigation.

**Method: **Data from surveys with graduates of the five medical faculties of Baden-Württemberg from the graduation years 2007/2008 (N=514) and 2010/2011 (N=598) were analysed.

**Results:** One and a half years after graduating 53% of those interviewed had completed their doctorate. When asked about their motivation for writing a doctoral thesis, participants answered most frequently “a doctorate is usual” (85%) and “improvement of job opportunities” (75%), 36% said that an academic career has been their primary motive. Less than 10% responded that they used their doctoral thesis as a means to apply for a job. The proportion of graduates working in health care is equally large among those who have completed a thesis and those who have not. Graduates who pursued a thesis due to scientific interest are also currently more interested in an academic career and recognise more opportunities for research. An implicit benefit of a medical thesis emerged with regard to the self-assessment of scientific competences as those who completed a doctorate rated their scientific competencies higher than those who have not.

**Discussion: **Although for the majority of physicians research interest is not the primary motivation for completing a doctorate, they might nevertheless achieve some academic competencies. For graduates pursuing an academic career the benefit of completing a medical thesis is more obvious.

## Zusammenfassung

**Einleitung:** Die Mehrheit der medizinischen AbsolventInnen in Deutschland promoviert, obwohl der Titel nicht zur ärztlichen Berufsausübung notwendig ist. Zur Frage, welchen individuellen Nutzen die Doktorarbeit aus Sicht der Promovenden hat, ist bislang nur wenig bekannt. Sie ist daher Gegenstand dieser Untersuchung.

**Methode: **Analysiert wurden Daten aus Absolventenstudien der Abschlussjahrgänge 2007/2008 (N=514) und 2010/2011 (N=598) der Medizinischen Fakultäten Baden-Württembergs.

**Ergebnisse: **53% der Befragten haben 1,5 Jahre nach Studienabschluss ihre Promotion abgeschlossen. Die stärkste Zustimmung zu Beweggründen, eine Doktorarbeit anzufertigen, erfahren die Motive „Promotion ist üblich“ (85%) und „Verbesserung der Berufschancen“ (75%). Dem Motiv, eine akademische Karriere anzustreben, stimmten 36% zu. Weniger als 10% der Befragten setzen ihre Promotionsarbeit als Strategie bei der Stellensuche ein. Der Anteil der AbsolventInnen, die in der Krankenversorgung arbeiten ist unter den Promovierten praktisch genauso groß wie unter den Nicht-Promovierten. Diejenigen, die für die Promotion stärker wissenschaftliche Motive angeben, sind auch aktuell stärker an einer akademischen Karriere interessiert und sehen für sich mehr Möglichkeiten wissenschaftlich zu arbeiten. Als impliziter Nutzen zeigte sich, dass die promovierten AbsolventInnen ihre wissenschaftlichen Kompetenzen signifikant höher einschätzen, als ihre nicht promovierten KollegInnen.

**Diskussion: **Obwohl die meisten MedizinerInnen eine Promotion nicht in erster Linie aus originärem Forschungsinteresse anstreben, erweitern sie dadurch dennoch ihre wissenschaftlichen Kompetenzen. Einen offensichtlicheren Nutzen hat die Promotion für diejenigen, die eine wissenschaftliche Karriere anstreben.

## Core Messages

1. Doctoral theses have an implied benefit for doctoral candidates in that they broaden their academic competence.
2. Around 35% of doctoral graduates wish to pursue an academic career.
3. A doctorate has an obvious benefit for all those intending to pursue an academic career, as they are more likely to grasp the opportunity of working within an academic field.

## Introduction

Every year much more than 6,000 physicians in Germany earn a medical doctorate [https://www.destatis.de/DE/Publikationen/Thematisch/BildungForschungKultur/Hochschulen/PruefungenHochschulen2110420127004.pdf?__blob=publicationFile]. 

This equals about 66% of all medical graduates of one year. Other studies also indicate that a medical doctorate seems to be the rule rather than the exception. Thus, one and a half years after graduation, some 80% of German medical graduates in 2005 and 2009 reported that they had started working on their doctorate or had already completed it [1]. Another survey [2] revealed that 75% of the graduates from 1997 had gained a doctorate ten years after their graduation.

Thus, the medical doctorate seems to be “an almost mandatory final degree in academic education.” [3]. This is all the more astonishing as the completion of a doctorate is no formal requirement for the pursuance of a career in medicine, and is generally an obligatory qualification stage in academic medicine only.

The status of the medical doctorate, its academic quality, and its collective and individual cost effectiveness is an intensively discussed subject. The German Council of Science and Humanities (Wissenschaftsrat) [4], for example, criticized the fact that doctoral theses in medicine often do not meet the academic standards set by other disciplines and stated that scope and academic quality are rather comparable to final degree papers e.g. master theses [5]. Adding to this, the German Research Foundation (DFG) called for a “departure from ‘pro forma’ research in order to avoid the waste of resources through “pseudo-academic” work without any far-reaching academic relevance” [6]. Another point of criticism is that clinical training is often extended when a doctoral thesis is being completed [7].

However, despite the severe criticism of doctoral theses in medicine, some arguments are also raised in their favor. With regard to the individual benefit for instance, the significance for the development of academic competence is stressed [8]. This includes, for example, the retrieval and interpretation of scientific evidence (e.g. research in scientific databases, critical evaluation of scientific studies). Further effects are assumed with regard to methodological and statistical knowledge [9].

Regarding the collective benefit, it is hoped to recruit junior researchers, in particular because the lack of well-trained academically active physicians has been deplored for many years [10]. Further benefits might also arise from the fact that in many areas doctoral candidates and their assigned sub-projects are already an integral part of many research endeavors.

Finally, other factors might also influence the importance of a doctoral degree, for example the prestige connected with a doctorate [http://www.zeit.de/campus/2009/01/pmc-titeltr-ger] or its formal relevance for certain career goals. Just how relevant a doctoral degree might be for the physician-patient relationship is open to debate. In common parlance, physicians are often referred to as “doctors” and addressed correspondingly. It is, however, questionable as to what extent the linguistic association physician/doctor reflects the expectation that physicians have indeed gained their title by completion of an academic thesis.

Various interests thus influence the importance and benefit of the medical doctorate. The concrete benefit for those who are writing, or have written a doctoral thesis has so far only partially been inferred [9], [11]. This study will therefore investigate the explicit (e.g. increased employment perspectives) and implicit (skills acquisition) benefits of doctoral theses. In addition, an overview of the duration and nature of the doctorate, its grading, and the motives for writing one will be given. This will involve the use of data from graduate surveys carried out within the medical faculties in Baden-Württemberg.

## Methods

### Questionnaire

Since 2008, the Centre for Evaluation of Teaching in Medicine at the Medical Faculty in Freiburg and the International Centre for Higher Education Research, Kassel (INCHER) have jointly carried out a yearly survey of medical graduates. The surveys take place anonymously and in compliance with data protection legislation. In addition, every two to three years the competence centre coordinates graduate surveys at all medical faculties in Baden-Württemberg. Alongside questions on the topics of study progress and conditions, acquisition of competencies (i.e. the Freiburg Questionnaire to Assess Competencies in Medicine, FKM [12]), search for employment and occupational orientation, the revised questionnaire for use in medicine also contains questions relating to doctoral studies. In the present study, those questions in particular were evaluated which are associated with the doctorate. They represent just a small part of all questions.

#### Sample

In order to verify the survey, data from two samples from graduates who completed their studies in 2007/2008 and 2010/2011 from the five medical faculties in Baden-Württemberg were analysed. The graduates were all interviewed around one and a half years after they had finished their studies. The survey was announced by mail. For the 2010/11 cohort, the letter of announcement already contained the link and access code for the online survey. In the subsequent course of the survey, paper questionnaires were used in addition. In the event of non-responses, a maximum of four reminders were sent out.

#### Statistical Methods

As well as χ^2^-tests, single factor variance analyses or t-tests for independent samples were predominantly carried out. Effect sizes (η^2^) were calculated which show how much overall variance can be resolved by the independent variables.

The statistical evaluations were carried out with the help of the statistics programme SPSS, version 20.

## Results

### Description of Samples

Table 1 (Tab. 1) shows the essential characteristics of the samples. With over 40%, the response rates were acceptable. The proportion of women was over 60%. On average, those interviewed were 28 years old when they graduated. Typically, they had all studied for 13 semesters. Between the samples of the survey and the basic population, there were no statistically significant differences regarding those characteristics given in the table. Thus the samples can be taken as representative.

If the information provided by the graduates from both cohorts is summarised, then 53% on average had completed a doctorate at the time of the survey. The time spent working on a dissertation was 21 months on average.

#### General Information on Doctorates

**Figure 1:**
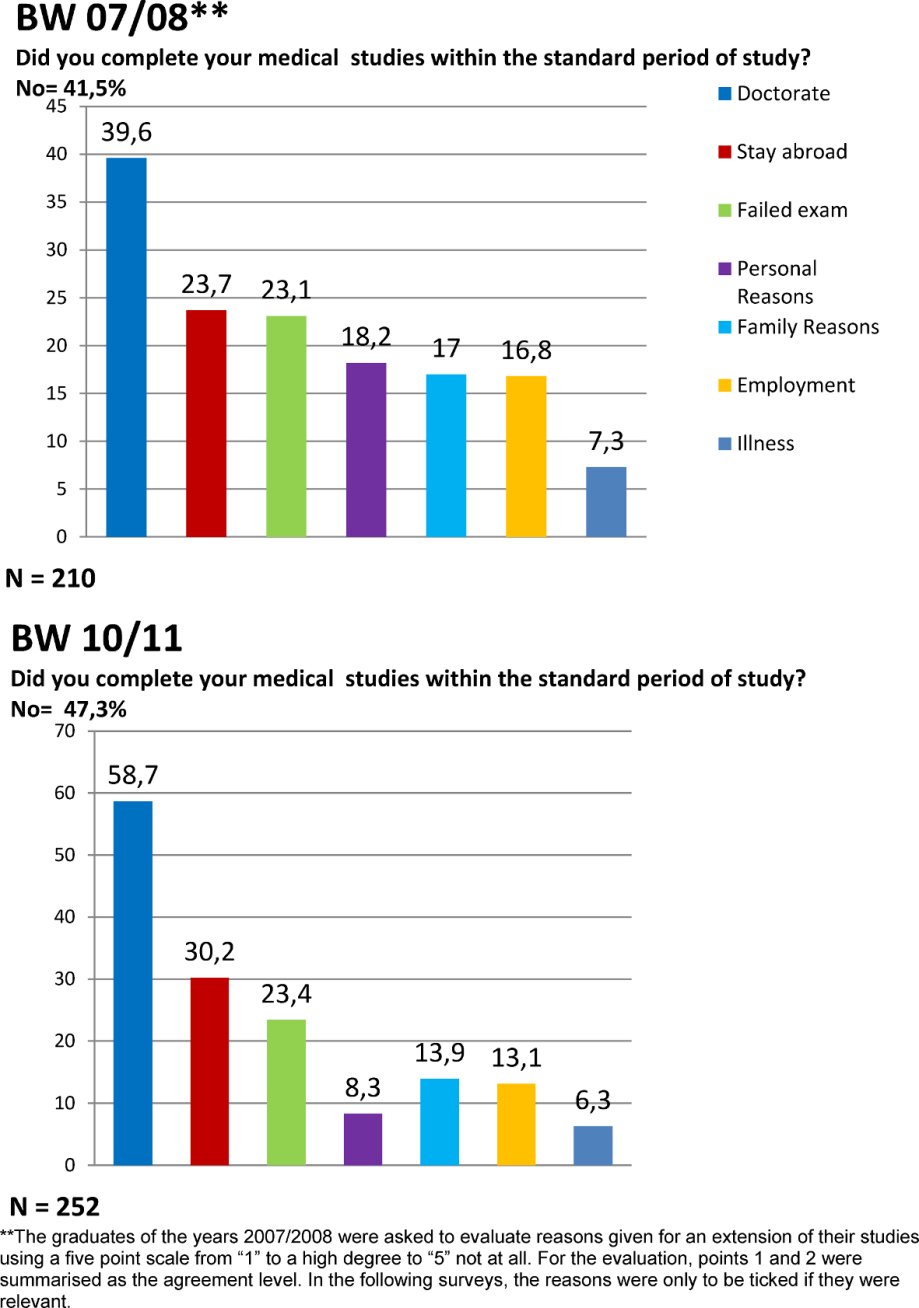
Reasons for the extensions of studies: results of the graduation years 07/08 und 10/11 to the question: Why did you study for longer than the standard period of study? (Multiple answers possible)

Figure 1 (Fig. 1) shows that 41.5% in 07/08 an 47.3 in 10/11 graduates studied for longer than the standard period of study. The most common reason given for this is the doctorate (39.6% and 58.7% respectively).

**Figure 2:**
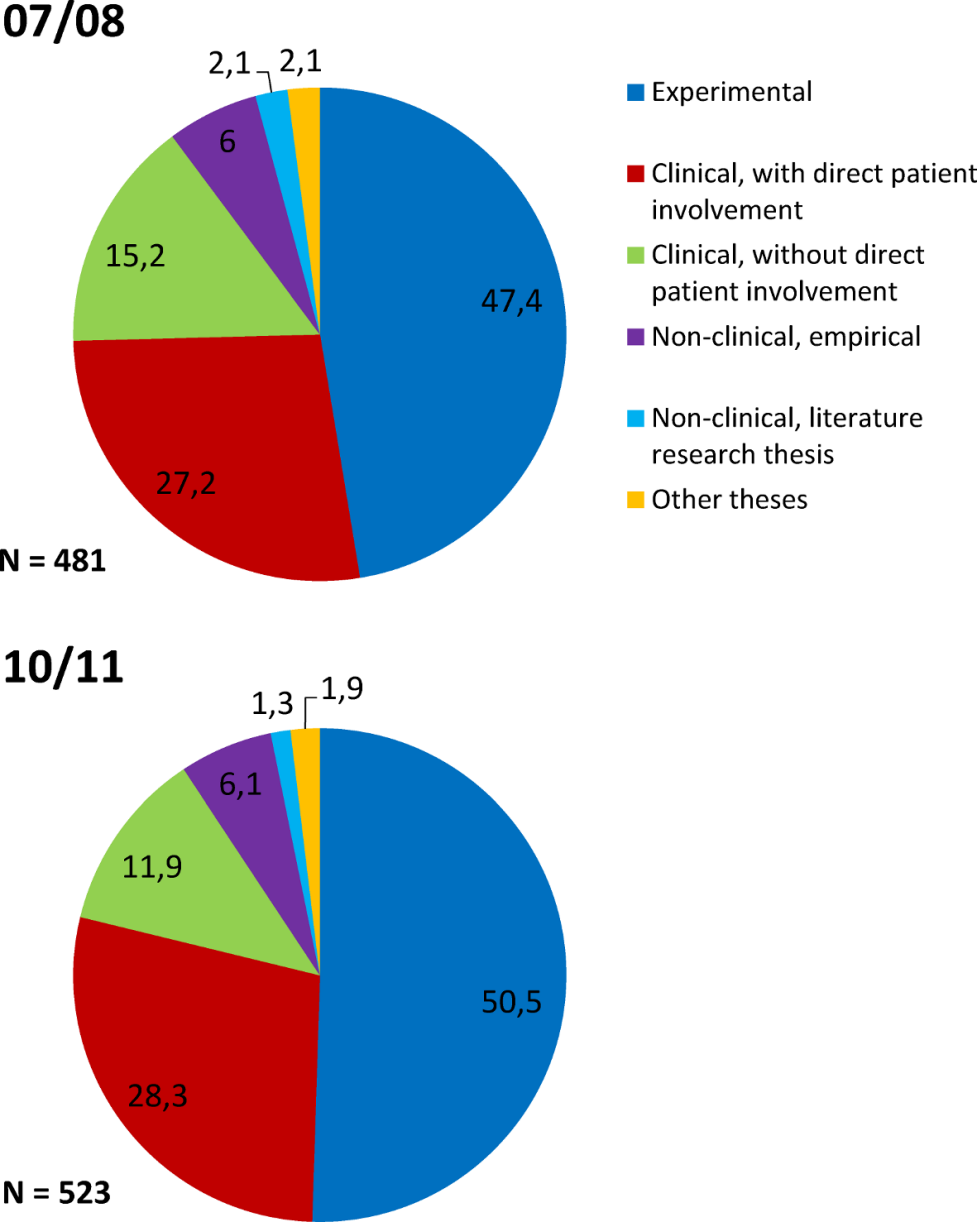
Types of doctoral theses. Percentages for the graduation years 07/08 und 10/11.

In Figure 2 (Fig. 2), the proportion of various types of doctoral theses are depicted. Experimental research studies are carried out most frequently; second most frequent are clinically-oriented studies with patient participation. These are followed by clinical studies where already existing data were analysed. Non-clinical, empirically-oriented topics are less frequently investigated, and only occasionally clinically-oriented literature studies. Further analyses show that experimental doctoral theses obtain the grades *summa cum laude* and *magna cum laude* more frequently than clinical studies with or without patient participation or literature studies (χ^2^_(2007/2008)_=76,578, df=12, p=.000, χ^2^_(2009/2010)_=93,709, df=12, p=.000). 

Overall, only about 7% of all theses were graded *summa cum laude*, and almost exclusively theses that had used experimental methodology were awarded that grade (around 90%).

#### Motivation for Obtaining a Doctorate

The specific motives for beginning a doctoral thesis were only recorded after the graduation year 2007/2008, and thus, the percentages are given for cohort 2010/2011 only. The most frequent motive given was “gaining a doctorate in the field is common” (85%). This was followed by motives such as “in order to improve my job opportunities” (75.3%), “for my own personal development” (68.7%), “because I want to research an interesting topic” (55.1%) and “in order to better fulfil my professional and occupational inclinations” (41.4%). 35.9% of participants stated that they “wished to pursue an academic career”. The least frequent motives were “in order to have the status of a doctoral student” and “in order to earn a higher income” (15.7% and 8.0% respectively).

#### Explicit Benefits of a Doctoral Thesis

**Figure 3:**
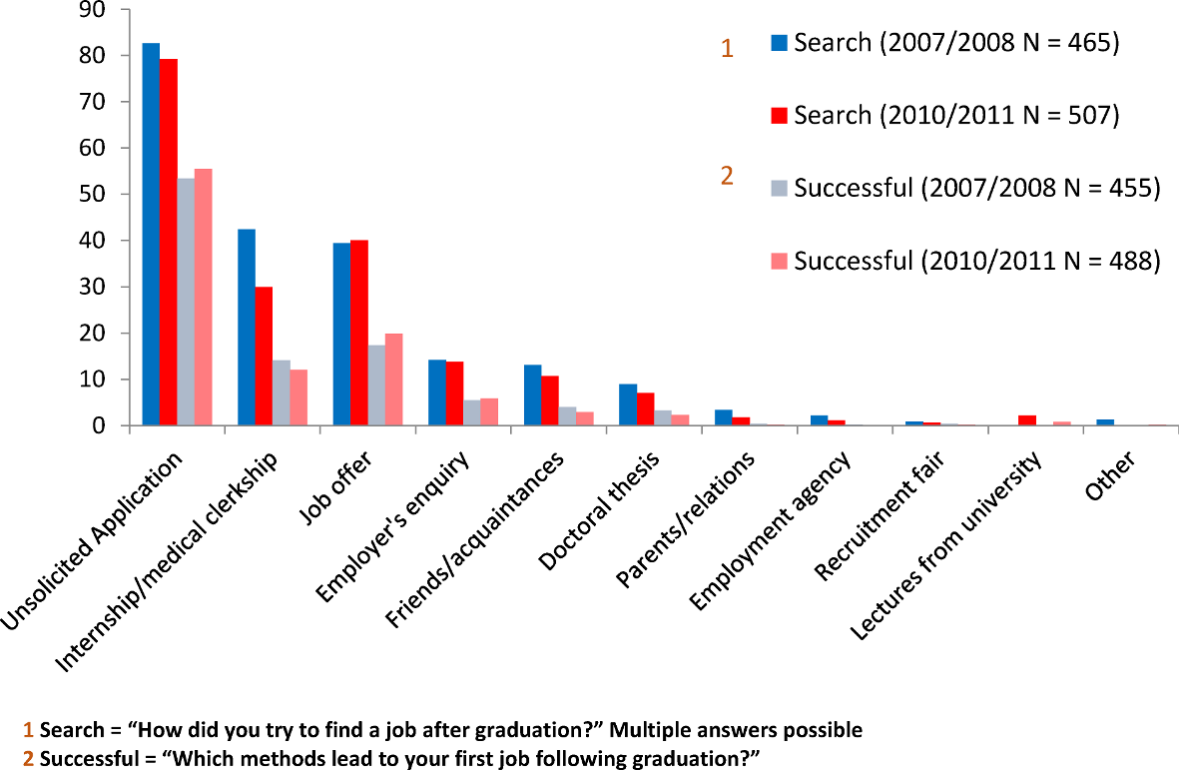
Job seeking strategies and their success (in percent). Results for graduation years 2007/2008 and 2010/2011.

In order to determine whether the doctoral thesis is relevant for the job market, the graduates were asked about the strategies they employed when first searching for employment. Subsequently they were asked which strategies had been successful. Figure 3 (Fig. 3) shows that unsolicited applications were favoured by the majority, and only a few male and female graduates applied for a job by referring to their doctoral thesis (9% and 7.1% respectively). The most successful strategy was the unsolicited application, whilst the doctoral thesis was less frequently given as a reason for employment. 

Analyses of the data taken from samples 2007/2008 show that the same proportion of graduates work in patient care regardless of whether they have completed a doctorate or not (94.8% vs. 95.7%; χ^2^_2007/2008_=.192, df=1, p=.662). Similarly, no significant differences in income between those with a doctorate and those without one could be determined (χ^2^_2007/2008_=21,163, df=18, p=.271).

The doctoral thesis is a precondition for an academic career. For this reason, a closer analysis was carried out with the sample of 2010/2011 to establish whether those who strongly agreed with the motivation of planning a career with the help of a doctoral thesis differed in other respects from those who showed little or no interest in an academic career.

Around one third of those questioned agreed that they were planning an academic career with their doctorate. These graduates are significantly more often (60.5% vs. 39.4%; χ^2^=85,316, df=2, p=.000) employed by university clinics than those who had less interest in an academic career. The opportunity of working in a scientific field is more important to them (F=119,862, df=2,345, p=.000, η^2^=0.41), and in their present professional situation they find more opportunities to engage in research than other graduates (F=28,670, df=2,332, p=.000, η^2^=0.15).

#### Implicit Benefits of a Doctoral Thesis

**Figure 4:**
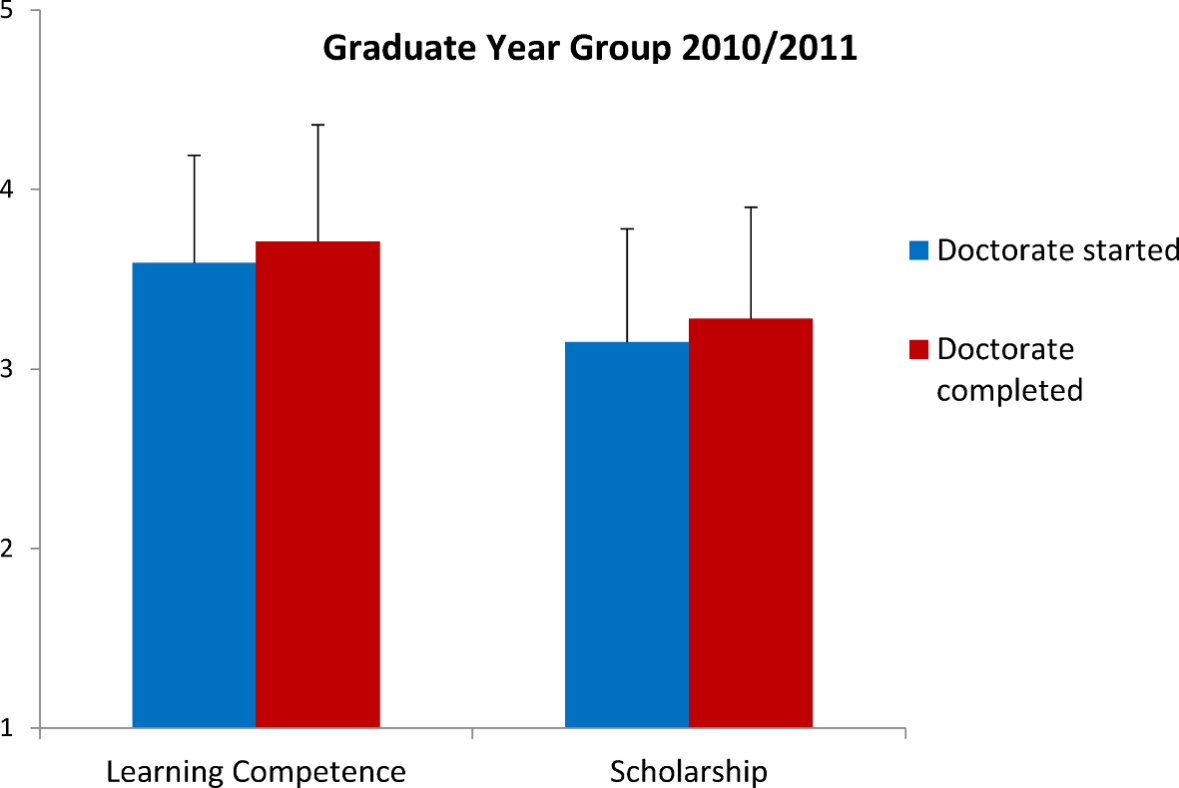
Retrospective evaluation of the existing level of learning competencies and scholarship at the end of studies, depending on whether the doctoral thesis has not yet been completed (n_LearnComp_=183, n_Scholarship_=178) or has been completed (n_LearnComp_=261, n_Scholarship_=256) for the graduation year 2007/2008 (1=not at all, 5=to a very great extent).

With regard to the implicit benefit of completing a thesis, self-assessments of competencies were analyzed. In retrospect graduates with a doctorat rated their learning competencies and research skills significantly higher than their colleagues without a doctorat (t_LearnComp_=-1.98, df 442, p=.048, t_Sholarship_=-2,123, df 432, p=.034; see also Figure 4 (Fig. 4)).

## Discussion

The present study inquired whether doctoral graduates of medicine have professional or personal advantages from their doctorate. The results provide no clear picture. In accordance with other surveys [2], [13], [http://www.zeit.de/campus/2009/01/pmc-titeltr-ger], over 50% of those surveyed had completed a doctorate one and a half years after qualifying. According to the graduates’ statements, they completed their theses within 21 months on average, making the doctorate the most frequent reason for a prolongation of medical education. Overall, students invest a great deal of effort to obtain a doctorate. Whether this commitment provides a corresponding benefit is revealed by further evaluations.

### Motivation for a Doctorate

In accordance with the results of another study [1], extrinsic motives for a doctorate are most common. In the present study, both the two most frequently named motives were “a doctorate is common” and “to improve job opportunities”. Motives related to educational interest or academic inclination were given less frequently. 36% agreed that pursuing an academic career was a motive. This result corresponds approximately to a recent survey of students from different faculties [12].

#### Explicit Benefit of a Doctorate

When we consider the indicators for the direct benefit of a doctoral thesis (“to improve my job opportunities”), then at this early stage in a career professional advantages are (still) hardly visible. A doctorate only very rarely has a decisive role for recruitment. Obvious differences in the employment situation of doctoral graduates and those without a doctorate cannot be determined: whether with or without a doctorate, approximately the same proportion of graduates work in health care and receive a comparable gross income. This last point is not surprising as a doctorate is not relevant for most pay scale classifications in public service; still it might have some more impact in privately run institutions or in business and industry. The career-related results are probably influenced by the current job situation for physicians which is quite comfortable: in some medical specialties, even university clinics experience difficulties filling posts immediately, so that even here a doctorate is no longer a necessary job requirement. However, it might be possible that a doctorate will become relevant only later in a professional career, when leading positions are being awarded, for example.

Despite the primarily pragmatic approach towards a doctorate, intrinsic motives do not appear to be unimportant. Indeed, two thirds of the participants stated that personal development was an important motive for a doctorate, and almost 50% named a specific research interest. These results strongly suggest that the high number of doctorates is mainly a result of the specific academic culture in medicine and also caused by status-related reasons. However, the additional effort for completing a thesis is not regarded a necessary evil, but rather as an opportunity to broaden personal horizons or to pursue an interest in science, even though these aspects may not be the primary motives for a doctorate. 

The high percentage of experimental theses, the direct benefit of which can be found above all in the improvement of scientific knowledge, is explained by the fact that experimental theses are those that are most commonly offered to students, and thus these types of studies are most frequently chosen regardless of the interest in a specific topic. It is therefore not surprising that the proportion of these types of theses is significantly higher than the number of graduates who will go on to pursue a scientific or academic career.

A doctorate appears to have an explicit professional benefit for those students who stated that an academic career was their primary motive for completing a thesis. For these graduates, the opportunity to work scientifically is more important than it is for their colleagues. Two indicators suggest that they are indeed more dedicated to academic work: on the one hand, more doctorates had already been completed in this group at the time of the survey; on the other hand theses in this group were significantly more often awarded a summa cum laude, so that a higher academic return can be presumed. In turn, these graduates work more frequently at university clinics and are more often involved in research activities in their present job. However, other reasons for the better grades of the experimental theses should also also be taken into account: such theses, for instance, might be more often included in externally-funded projects (e.g. by the DFG), and thus might be more intensively supervised. Nevertheless, our data suggest that this type of theses is more likely to attract those with a greater interest in research.

#### Implicit Benefit of a Doctorate

The indicators for an indirect, implicit benefit show that the graduates achieve their goal of personal development by completing a thesis: those who have already completed a doctorate consider above all their academic competence to be significantly higher than those who have not yet completed a doctorate. 

Thus, a multifaceted picture of the medical doctorate arises: the majority of medical students decide to complete a doctorate, as they still feel it is expected of them; in doing so, however, academic interests and personal self-improvement do also become important. Moreover, for those whose primary interest is academic, frequently a doctorate seems to be the first step towards an academic career. Furthermore, those who are academically motivated not only seem to write the best theses, but also to complete them more rapidly. Ultimately, in the majority of cases a doctorate appears to result in a personal gain in academic competence.

#### Limitations of this Study

The results of the study are based on survey data from two samples of graduates who studied at the five medical faculties in Baden-Württemberg. The results are consequently not necessarily valid for other faculties. Moreover, this is a retrospective survey so distortions cannot be ruled out. Finally, in reference to academic competence, it has to be pointed out that these were self-assessments.

#### Outlook 

In particular, those aspects that deal with the explicit benefit of a doctorate might be verified by panel surveys five and ten years after graduation. Thus it could be determined to what extent a high-end doctorate might effectively form the cornerstone of an academic career.

## Conclusions

The view of those graduates taking part in the survey supports the notion that a medical doctorate appears primarily to be an “unofficial” degree. 

Despite the fact that under the present labour market conditions, a doctorate has virtually no effect on employment opportunities or salary, at least at the beginning of a career in medicine, the assumption of professional advantages is a major motive for a doctorate. In light of the consistently high number of doctorates, it can be presumed that it is impossible to fulfill the oft-repeated demand that every doctorate should provide a qualitative or independent contribution to science in the first place. However, our data also show that this verdict is not true for all doctoral candidates. Those who are primarily academically motivated, and who wish to pursue an academic career, more frequently produce theses that meet the indicated academic standards that in turn give them the opportunity to pursue a career in science. Whilst a medical doctorate primarily serves to gain distinction, at the same time it also fulfils the function of self-recruitment for the medical science system. Whether this last point occurs on a sufficient scale cannot be decided here. However, in light of the motives documented here, it appears to be doubtful whether the proportion of those who decide to pursue a career in academia could easily be increased.

## Acknowledgements

We would like to thank our cooperation partner, the Competence Network “Teaching in Medicine Baden-Württemberg” [http://www.medizin-bw.de/Kompetenznetz_Lehre.html] for their excellent cooperation.

## Competing interests

The authors declare that they have no competing interests.

## Figures and Tables

**Table 1:**
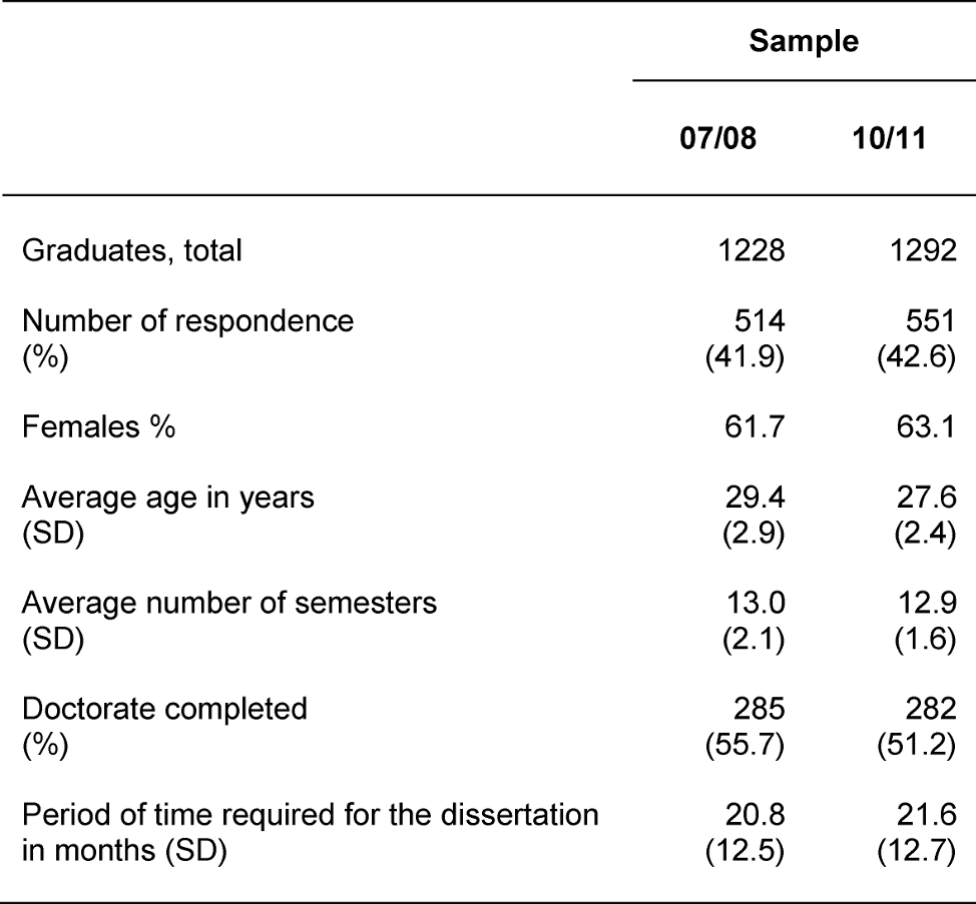
Sample Descriptions
